# Emerging interdependence between stock values during financial crashes

**DOI:** 10.1371/journal.pone.0176764

**Published:** 2017-05-25

**Authors:** Jacopo Rocchi, Enoch Yan Lok Tsui, David Saad

**Affiliations:** 1 Nonlinearity and Complexity Research Group, Aston University, Birmingham, B4 7ET, United Kingdom; 2 Department of Physics, The Hong Kong University of Science and Technology, Clear Water Bay, Kowloon, Hong Kong, China; University of Warwick, UNITED KINGDOM

## Abstract

To identify emerging interdependencies between traded stocks we investigate the behavior of the stocks of FTSE 100 companies in the period 2000-2015, by looking at daily stock values. Exploiting the power of information theoretical measures to extract direct influences between multiple time series, we compute the information flow across stock values to identify several different regimes. While small information flows is detected in most of the period, a dramatically different situation occurs in the proximity of global financial crises, where stock values exhibit strong and substantial interdependence for a prolonged period. This behavior is consistent with what one would generally expect from a complex system near criticality in physical systems, showing the long lasting effects of crashes on stock markets.

## Introduction

Financial markets in general and the drivers for market crashes in particular have been intensively investigated by the physics community in recent years. The interest in markets’ behavior stems from a number of different reasons relating both to the narrow financial interests of shareholders and investors and, arguably more importantly, to the devastating impact financial turmoil may have on national economies, leading to harsh social consequences and societal unrest. Moreover, the observation of a sudden and dramatic collapse in complex systems intrigues the scientific community due to its resemblance to collective rearrangement in physical systems at critical points. This problem is at the core of the emerging field of econophysics [1–3], which borrows mathematical and physical tools such as random matrix theory [4, 5], clustering analysis [6], extreme and rare events [7], agent based models [8] and network theory [9], to name a few, to tackle the complexity of economical and financial systems. Network theory provided a framework for analyzing economic structures [9–12] from the perspective of complex systems [13], and is rooted in a much earlier search for structures in financial markets [14]. The success of this approach is partly due to shortcomings of existing economics-based theories in addressing the complexity of financial systems, and partly because many phenomena such as financial bubbles, herding, contagion and crashes found a natural interpretation in physical models that involve multi agents, collective behavior, influence spreading and phase transitions.

Phase transitions are sudden reorganizations of the system occurring when an external parameter, such as the temperature, is tuned to a critical value. Moreover, at this critical point, the system is scale-invariant, leading to a power law behavior of the observables whose critical exponents may be studied using renormalization group and scaling theory [15]. A similar symmetry, discrete scale invariance [16], has been shown to give rise to log-periodicity of prices in the proximity of financial collapses [17]. Switching trends in financial markets have been analysed in [18], where first order phase transitions, like those occurring in low temperature Ising models when tuning an external magnetic field, have been proposed as for explaining herding behaviour in the proximity of crises. Important contributions to the analogy between financial crashes and phase transition came also from the study of ecological and climate systems. In particular, a large effort has been devoted to studying the precursors of collapses [19–21], showing that crashes are usually anticipated by a loss of resilience in the system; in other words, when approaching a critical point, perturbations take more time to be reabsorbed and are more likely to propagate. This picture is consistent with that of physical systems near the critical point of a phase transition, where the cross correlation between the fundamental degrees of freedom of the systems is very large and the system exhibits high susceptibility.

The study of correlations in financial networks has started a couple of decades ago and led to the development of several effective algorithms for extracting the underlying network topology [9, 10, 22–25]. Their hierarchical structure could then be used to identify groups of stocks in terms of the corresponding economical sectors. These works originally focused on same-time correlations and only recently have been generalized to deal with the concept of causality [26] in financial data [27]. The study of directed influences, aiming at predicting future prices, is interesting for investors for maximizing their returns; however, it also has the potential to forecast the macroscopic behavior of markets. This objective is highly ambitious, but predicting the behavior of macroscopic properties from observation and modeling is at the heart of statistical physics and is of theoretical interest on its own right. Attempts in this direction has been made using Granger causality [28] and information theoretical methods such as mutual information and Transfer Entropy (TE) [29], which outperform simple retarded correlations in capturing non-linear influences [30, 31].

In this paper we generalize existing information theoretical approaches to study temporal interdependencies between financial indices in a time period of several years. More precisely, we analyze interdependencies between stocks of the FTSE 100 companies, which includes the 100 largest companies (in terms of market capitalization) listed on the London Stock Exchange, from 2000 to 2015. Exploiting the physical intuition that financial crashes may be anticipated by periods of large susceptibility, we use this method to investigate directed influences among the corresponding index constituents looking for a similar behavior, with the limited resolution of daily stock values. Most of the literature in this area relies on intra-days influences since it is commonly believed that traders are well informed when making their decision, so that directed correlations and influences can be detected only at short time scales. This belief is an interpretation of the “efficient market hypothesis” (EMH) [32], according to which there is no hope to predict and outperform the market. Strictly speaking, this is called “strong market efficiency” and originated from the faith in the ability of the market to quickly process all the available information and to push prices towards their fundamental values [33]. As we will discuss, it is not always true and these situations are usually described in terms of “weakly” efficient markets. In fact, analyses of daily stock prices reveal that they do not behave as martingales [30, 34] and that during the crash of 2008 price movements have been highly correlated [35, 36]. Similar problems have been studied also in [37, 38] where it has been noticed that the aggregated correlation between stocks sharply increases after a period of market downtrend. We investigate this behavior analyzing moving periods of roughly two years each and find results that support the common view according to which little information can be extracted from the past to predict future values at daily time scales. However, employing directed information theoretical methods inspired by TE, we measure a boost in the overall information flow between index components in periods corresponding to the crash of 2008 and the Eurozone debt crisis of 2010-2012.

The paper is organized in the following way. The second section focuses on the materials and methods used, and is divided to three subsections. The first introduces the information theoretical methods, which are commonly used for extracting direct influences, the second provides details on the dataset and the third explains the null model used to validate our analysis. The third section contains the results and the following ones provide a discussion and conclusion. Further details on the methods are provided in the Supporting Information.

## Materials and methods

### Measuring influences

In order to measure the influences among stocks we used an Information theoretical tool, which follows from a generalization of the Symbolic Transfer Entropy (STE) [39]. TE and STE are powerful methods able to measure the amount of information flow between time series and thus can be used to reconstruct the network of influences between components of a complex systems. Some of the most interesting cases where they have been successfully employed include network reconstruction of functional areas of the brain [40, 41], the study of social phenomena [42] and the influence of social networks on financial markets [43]. They have also been used in finance to analyze the relations between indices [44, 45] and components of indices [31].

Transfer entropy evaluates the information gained on future values of a time series *X*(*t*) by observing past values of another time series *Y*(*t*) in addition to the past values of *X*(*t*), relying on estimating the probabilities of occurrences of time series values. For real valued time series this is more difficult but STE, making use of symbolization, provides an effective solution to this problem. A symbol of *k*−literals of the time series *X*(*t*) at time *s* is obtained by reordering the last *k* values of the time series at time *s* (i.e. {*x*_*s*−*k*_, …, *x*_*s*−1_}) in an ascending order. By generalizing the time step from 1 to an integer *δ* we transform the data to a set of *k*−dimensional symbols at times *s* + *δ*, denoted by ${\hat{x}}_{s+\delta }^{k}$. The role of the time scale *δ*, which reflects the inherent effective delayed interaction between components will be investigated later. A more formal definition as well as further details on the corresponding information theoretical measures are provided in the Supplementary Information.

In this work, we evaluate the influence of time series *Y*(*t*) on *X*(*t*) by computing the following quantity

$$\begin{array}{c} T_{Y\to X}=\sum_{{\hat{x}}_{t+\delta }^{k+1},{\hat{x}}_{t}^{k},{\hat{y}}_{t}^{k}}p\left({\hat{x}}_{t+\delta }^{k+1},{\hat{x}}_{t}^{k},{\hat{y}}_{t}^{k}\right)\log\left(\frac{p\left({\hat{x}}_{t+\delta }^{k+1}|{\hat{x}}_{t}^{k},{\hat{y}}_{t}^{k}\right)}{p\left({\hat{x}}_{t+\delta }^{k+1}|{\hat{x}}_{t}^{k}\right)}\right)\;. \end{array}$$ (1)

This is the Kullback-Leibler divergence between the probabilities $p({\hat{x}}_{t+\delta }^{k+1}|{\hat{x}}_{t}^{k},{\hat{y}}_{t}^{k})$ and $p({\hat{x}}_{t+\delta }^{k+1}|{\hat{x}}_{t}^{k})$, averaged over past symbols. Alternatively it can be viewed as the difference between the conditional entropies of the two probabilities. If *Y*(*t*) contains no information about *X*(*t*) this measure is zero. Practically, due to the noisy nature of the data this never happens and one finds non zero values even when the two systems do not interact. This calls for the introduction of a null model to extract the genuine underlying behavior from the dataset; this will be discussed in detail later on. Finally we would like to emphasize that our measure aims at predicting (*k*+1)-dimensional symbols by looking at *k*-dimensional historic symbols. This is a slight modification of the STE measure, where *k*-dimensional symbols are predicted by looking at *k*-dimensional historic symbols. Further details are provided in the Supplementary Information.

### Dataset

We collected financial time series data of the component stocks of FTSE 100 from 3 January, 2000 to 15 May, 2015 (around 4000 trading days), available from Yahoo! Finance [46]. Data is freely available at [47]. Clearly, the FTSE 100 index is continuously changing over time as companies migrate in and out of the index [48]. To mitigate the effect, rather than taking into account individual component changes, we considered the list of the FTSE 100 constituents in May 2015, and analyzed the related time series, tracing them back to January 2000. About 60% of the stocks that have been included in the May 2015 FTSE 100 index were already included in the index in 2000. Discarding short-lived stocks, we labeled the remaining *N* = 97 stocks from 1 to 97 in ascending alphabetical order of their ticker symbol. These time series have a time resolution of one (trading) day and we focused on the closing prices. Instead of looking at the whole time series, we analyze time windows of Ω = 500 days. This time window is being shifted by *ω* = 25 days across about 4000 trading days for which we have data, investigating the time evolution of the network structure. Since the stationarity hypothesis, useful in estimating the probabilities Eq (1), is unlikely to hold for long periods, studying shorter time windows would help in getting more reliable estimates. We process the time series linked to each stock in order to obtain the geometric returns *r*(*t*) at the time scale *δ*:

r(t)=log[p(t+δ)]-log[p(t)], (2)

where *p*(*t*) is the price at time *t* (closing price on day *t*). In each time window, we compute the information flow between time series at different *δ* values using Eq (1); while log-ratios with large *δ* values are expected to carry little or no information we will show that also log-ratios with small *δ* values do not, in certain periods. To further control errors and ensure that the stocks considered had existed for long enough to give rise to meaningful influences, we restrict the computation of *T*_*Y* → *X*_ to cases where the number of days the considered pair of stocks {*X*(*t*), *Y*(*t*)} have in common is at least 80% of the time window Ω.

### Surrogate dataset

To validate our results and eliminate spurious instances of entropy transfer, we construct a null model of non interacting components. This may be done in several ways [31, 42, 44, 45], ranging from a random reshuffle of the original time series to more refined methods [49, 50]. A simple reshuffling of data, while clearly destroying the interdependence among different time series, also destroys the single time series structure. The null model we use, based on the theory of surrogate data [51], does allow one to preserve the spectral properties of the original spectrum in spite of the randomization. Under the assumption that the single time series structure can be effectively represented by the power spectrum of the signal, a general time series *X*(*t*) can be randomized via the generation of the time series

$$\tilde{X}\left(t\right)=F^{-1}\left[X\left(k\right)e^{i\phi (k)}\right]\;,$$ (3)

where *X*(*k*) is the Fourier transform of the original signal, X(k)=F[X(t)], and *ϕ*(*k*) is a random phase attached to each Fourier component such that *ϕ*(−*k*) = −*ϕ*(*k*), so that $\tilde{X}\left(t\right)$ is real. The series $\tilde{X}\left(t\right)$ is thus a randomized version of *X*(*t*) but having the same power spectrum. We construct the null model by randomizing the original time series of the closing prices. Then, for a given *δ*, we process these time series to obtain random returns using Eq (2) and compute the influences between the surrogate time series using Eq (1). We compared the information flow in our original dataset and the null model to identify true information from noise since the latter does not contain genuine information flow between series.

## Results

We analyzed the evolution of the network of influences between stocks in each of the (about) 140 time windows indexed by *w*. We associate a value *I*(*X*, *Y*) ∈ [0, 1] to each directed link {*X* → *Y*} in order to measure the amount of genuine information flow from *X*(*t*) to *Y*(*t*),

$$I\left(X,Y\right)=\frac{1}{e^{2a(r\left(x\right)-r^{*})}+1},$$ (4)

where the parameters *a* and r* are set to 100 and 0.03, respectively. From a qualitative point of view, *r*(*x*) represents the probability that the information flow *x* = *T*_*X* → *Y*_ value calculated from the real dataset has been obtained at random. Further details on this definition are provided in S1 File, see Eq (13). The quantities *I*(*X*, *Y*) are supposed to vary slowly from one time window to the next, say *w* to *w* + 1; conversely, the parameter *ω* = 25 that controls the shift between consecutive time-windows, may be too large or our results may not be sufficiently stable with respect to small changes. To address this issue we introduced the quantity

$$D\left(w\right)=\frac{1}{N}\sum_{i=1}^{N}\left|\sum_{j=1}^{N}I_{w+1}\left(X_{i},X_{j}\right)-I_{w}\left(X_{i},X_{j}\right)\right|\;,$$ (5)

where, the expression within the absolute value sign is the change in the genuine information flow originating from stock *i* in two consecutive time windows.

**Fig 1:**
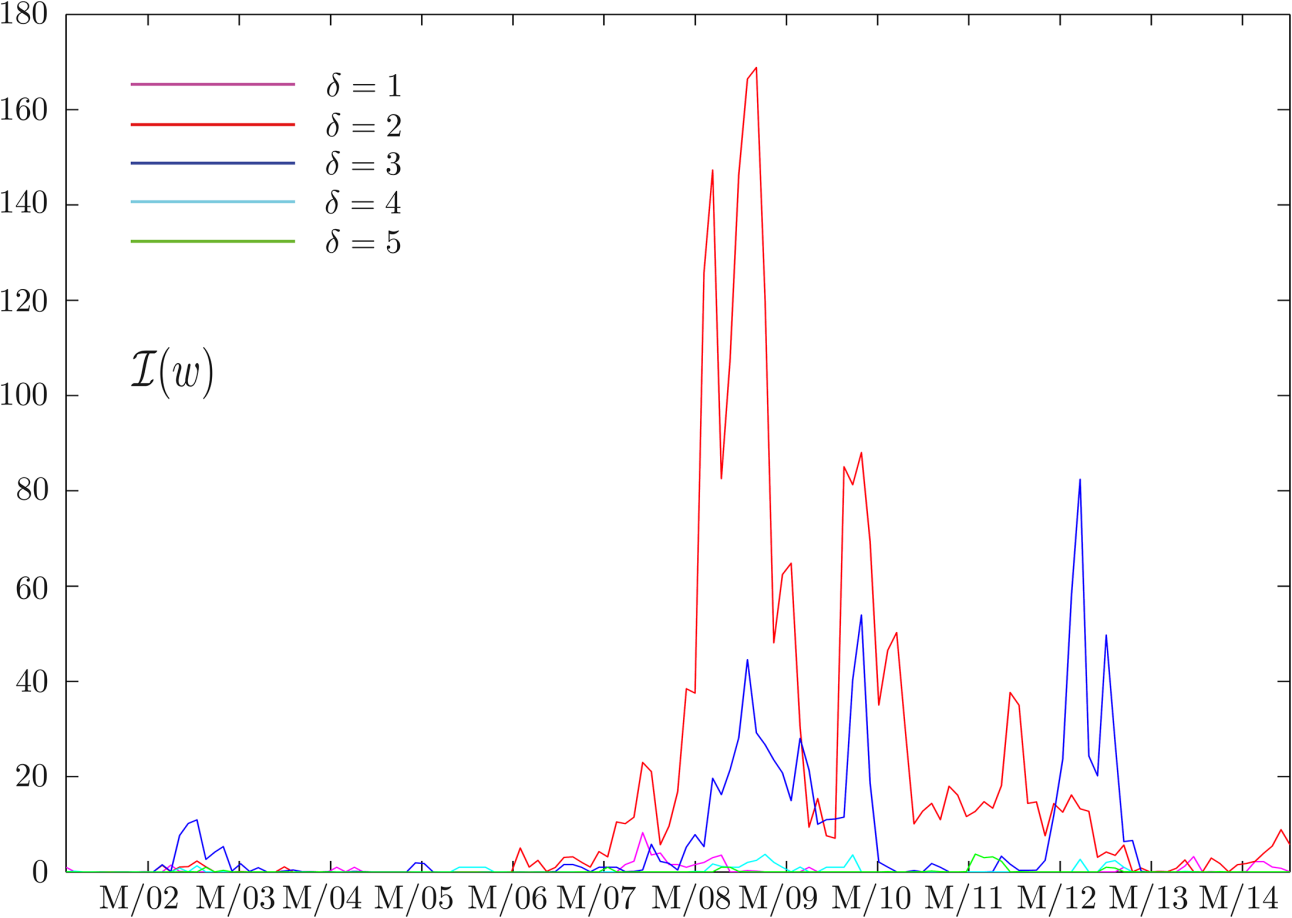
Total information flow as a function of time. The behavior of the total information flow I(w), defined in Eq (6), at different time scales *δ*. Each time window *w* is 500 days long. The date associated to each *w* is the middle of the time window considered. The *x*-axis tick marks represent the first of March of every year. While at short time scales (less than 3 days) we observe a peak around the two major financial crises of the last decades, this effect fades away as *δ* increases. Interestingly, the results at *δ* = 2 carries much more information than those at *δ* = 1.

**Fig 2:**
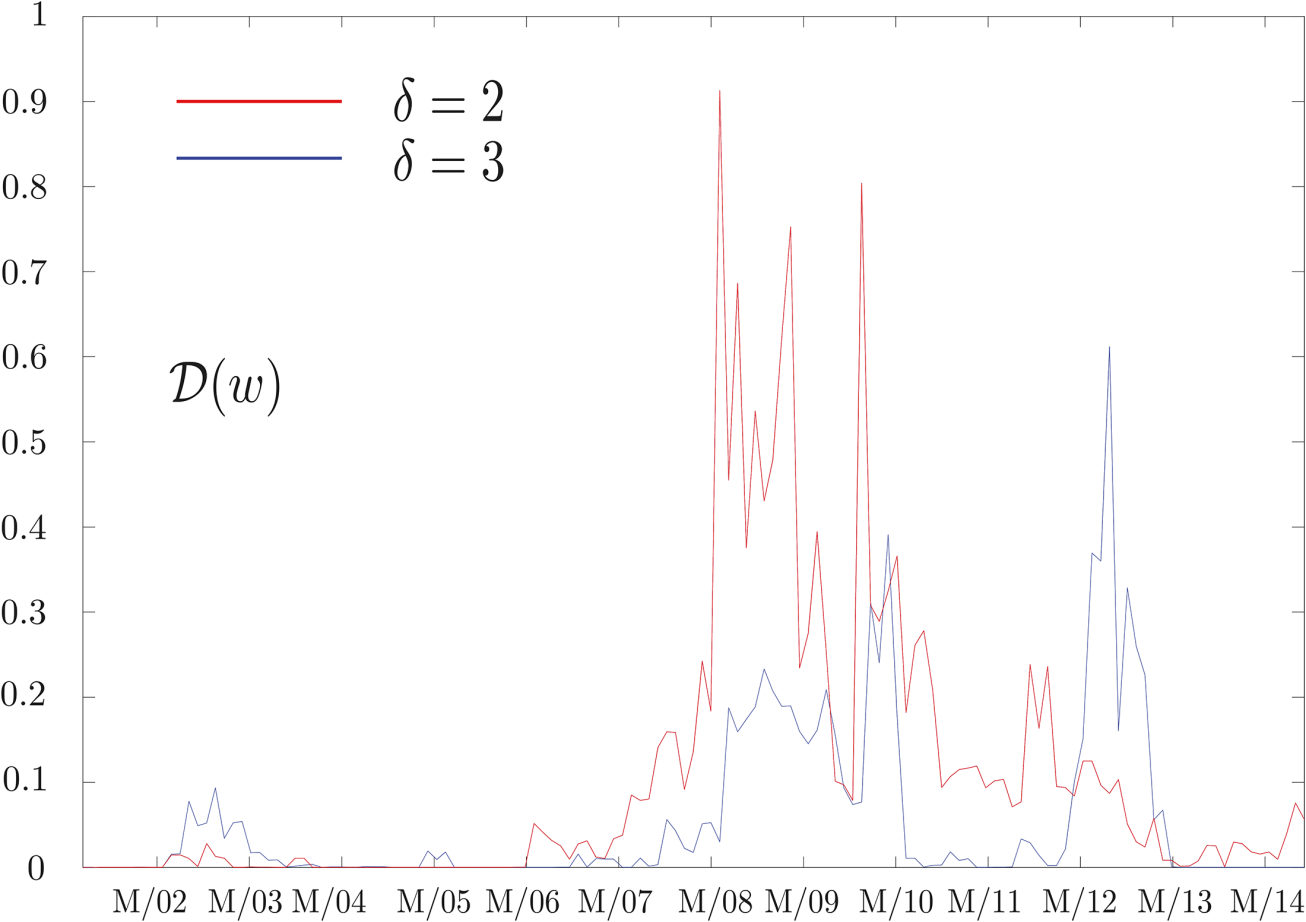
Network evolution. Plot of D(w), the information flow in two consecutive time windows, defined in Eq (5), at time differences *δ* = 2 and *δ* = 3. Each time window *w* is 500 days long. The date associated to each *w* is the middle of the time window considered. The *x*-axis tick marks represent the first of March of every year. This quantity measures the evolution of the detected structure of influences. We observe a smooth behavior, meaning that structures in consecutive time windows are similar, except for during crises where more pronounced market readjustment take place.

To estimate the *total* information flow, we introduce the quantity

$$I\left(w\right)=\sum_{X,Y}I\left(X,Y\right)\;,$$ (6)

and study its behavior as a function of *δ* and *w*. While the results for large *δ* confirm our expectations that no information flow can be detected, the results obtained at small *δ* as a function of *w* are much more interesting. In particular, analyzing geometric return time series by setting *k* = 2 in Eq (1), we obtain the results shown in Fig 1. At small values of *δ* this quantity sharply peaks around the financial crisis of 2008, while this effect fades away as *δ* increases. We also notice that a similar behavior is observed during the period of the Eurozone debt crisis between 2010 and early 2013, and then again fades away as *δ* increases. The robustness of our results can be checked analyzing the behavior of D(w). As can be seen in Fig 2, this quantity, representing the information flow in two consecutive time windows, is close to zero most of the time and peaks at a value smaller than 1. This confirms our assumption that the structure of influences is evolving smoothly. Moreover, it drops to zero when *δ* increases, since there is no information flow in any windows.

Finally, by manipulating the matrix *I*(*X*, *Y*), it is possible to probe a more detailed structure of the influences, introducing the information directionality flow

$$\Delta_{n}=\sum_{j\neq n}I\left(X_{n},X_{j}\right)-\sum_{i\neq n}I\left(X_{i},X_{n}\right)\;.$$ (7)

**Fig 3:**
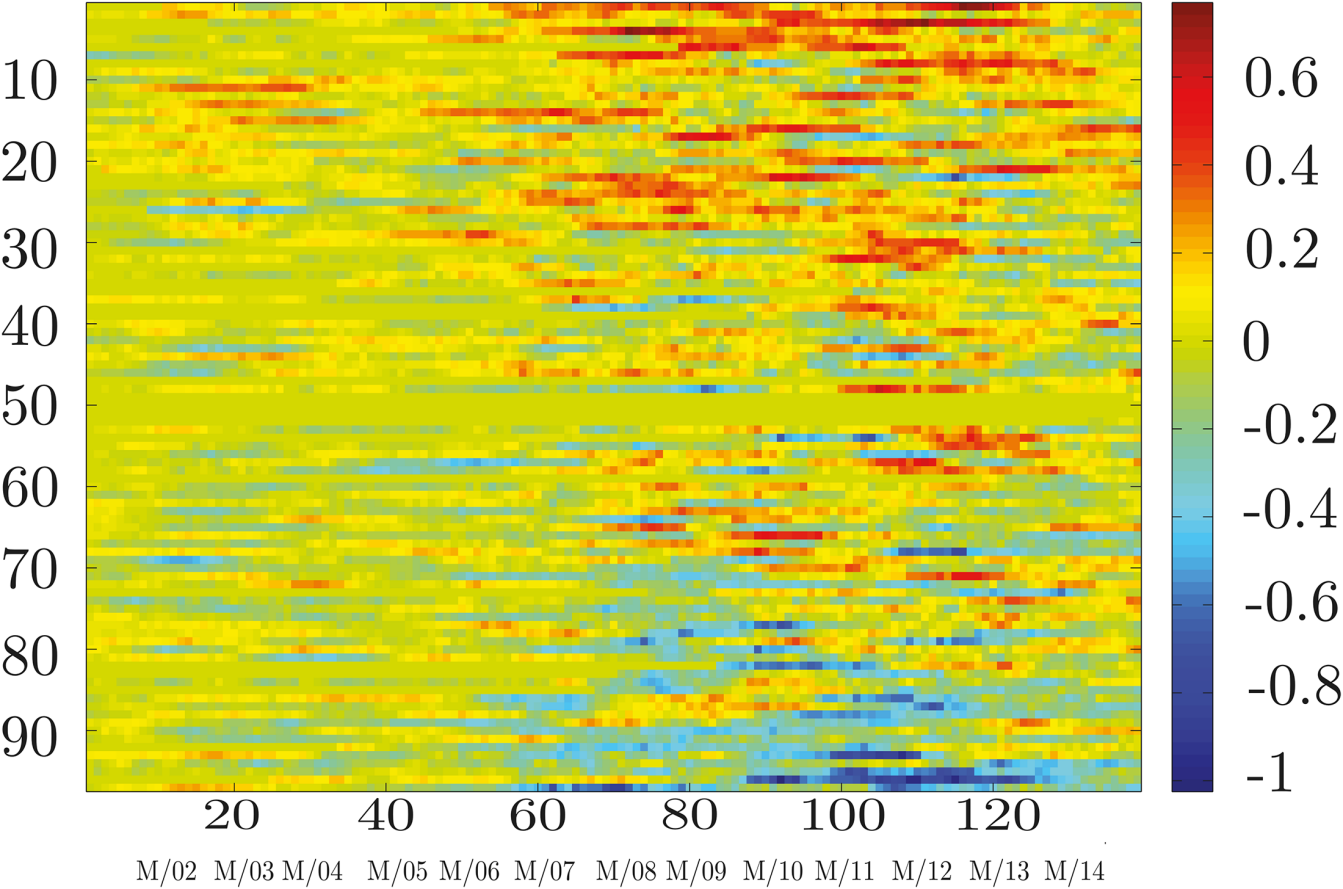
Information directionality flow. For each component *n*, we evaluate the information directionality flow Δ_*n*_, defined in Eq (7), measuring how much the component has influenced (or has been influenced by) the market. Positive values are associated to lead effects. The horizontal axis refers to the window time index *w*. The vertical axis refers to the component index. It is interesting to see how strength and directionality of influences become clearer and more emphasized at time of financial crises. A closer look at these values is provided in Figs 4 and 5.

**Fig 4:**
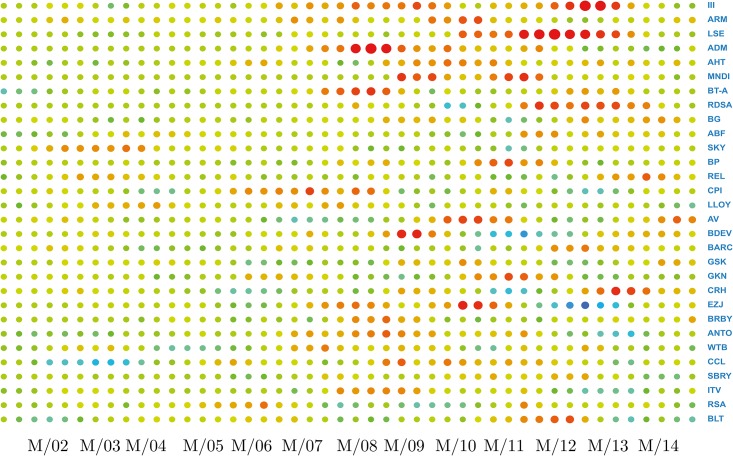
Directionality leaders. To identify more clearly leading stocks in terms of their effect on others, we present the information of Fig 3, but focussing on the 30 components with the *largest* directionality flow values. For the sake of clarity each time tick has been obtained by averaging three consecutive time windows. So we have about 45 different ticks rather than the original 140 time windows.

**Fig 5:**
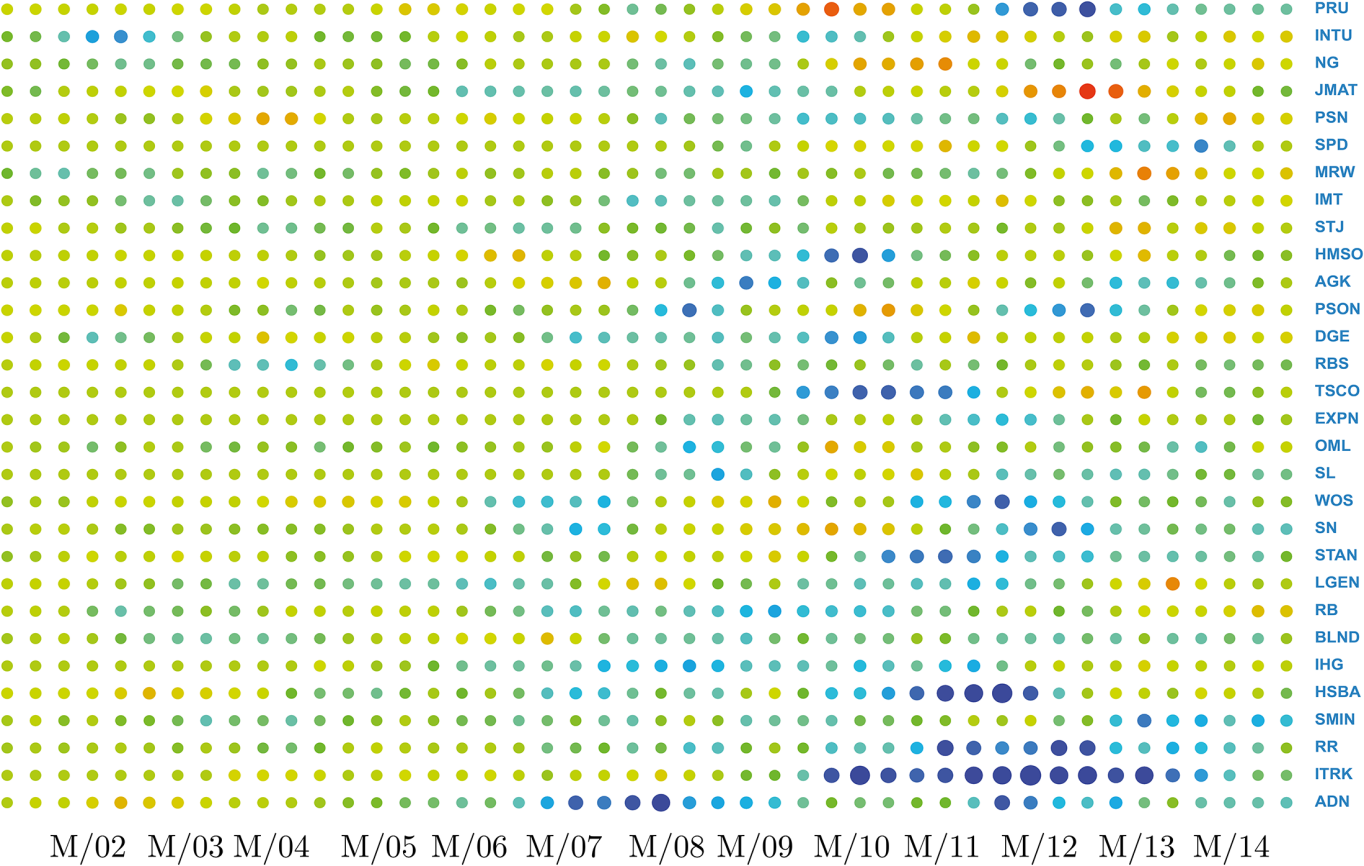
Directionality followers. To identify more clearly stocks led by the market, we present the information of Fig 3, but focussing on the 30 components with the *smallest* directionality flow values. For the sake of clarity each time tick has been obtained by averaging three consecutive time windows. So we have about 45 different ticks rather than the original 140 time windows.

The first term is a summation across columns, measuring the total information flow originating from the stock *n*, while the second is a summation across rows, measuring the total information flow directed toward *n*, originated from other stocks. The difference between these two terms provides a measure of whether the stock *n* is influencing the market (Δ_*n*_ > 0) or is being influenced by the market (Δ_*n*_ < 0), and by how much. A plot of the stock’s directionality measures with time provides knowledge about how the stock’s role in the market has been evolving, as shown in Figs 3–5. This picture also provides another check on the slow evolution of the matrices *I*(*X*_*i*_, *X*_*j*_) during several time windows.

## Discussion

**Fig 6:**
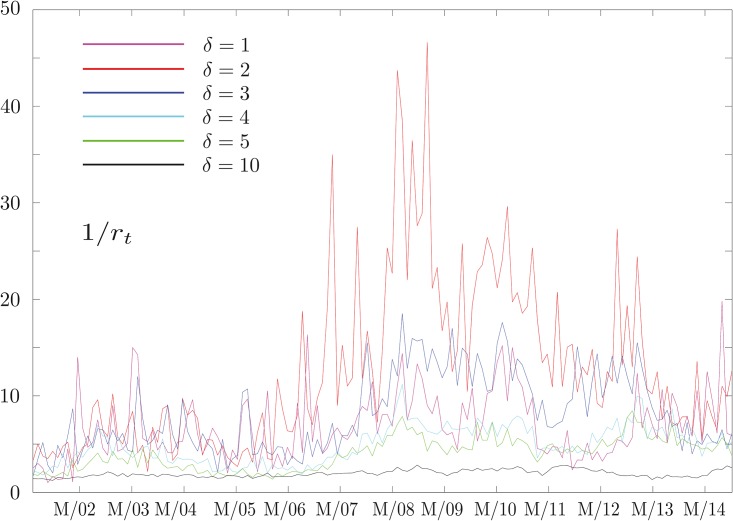
Thresholding method. We compute the ratio of the number of directed links detectable in the real and surrogate datasets. We define this ration as 1/*r*_*t*_. The threshold used is 0.03 but similar qualitative results are obtained for other values. We see that as *δ* increases this ratio approaches 1, while at short time scales it resembles the results of Fig 1.

To validate the results obtained using a different approach we employed another method to investigate the data. This method is based on extracting all the pairs {*X*, *Y*} for which *T*_*X* → *Y*_ is larger than a given threshold and then, using the same threshold, extracting all pairs of a *surrogate dataset* of time series for which the same condition is satisfied. Finally, we compare the number of links extracted in the two cases. The surrogate dataset, obtained following the protocol outlined above, does not include genuine interactions between its degrees of freedom at any of the time windows considered and we do not expect it to reproduce the patterns found in Fig 1. More specifically, we do not expect to observe an abrupt increase in the number of links around crises. Fig 6 shows that such significant increases are not observed for the surrogate data and therefore these are genuine phenomena of the real dataset.

We would like to point out that while we mainly discuss results obtained for *k* = 2, we also carried out the analysis for larger *k* values. Computing the matrices *T*_*Y* → *X*_ for larger *k* values requires much more time; additionally we were unsuccessful in extracting meaningful information *I*(*X*, *Y*) even for *k* = 3. This is disappointing but is confirmed by other works [44] studying the TE measure Eq (1) by varying the parameter *k*, where it has been shown that large values of *k* provide uninformative results. A possible reason is the signal-to-noise ratio, which is very small already for *k* = 2 and is presumably lost in more complicated models of influences. Moreover, when increasing *k* at a fixed Ω, we notice that the quality of the probability estimation in Eq (1) decreases.

**Fig 7:**
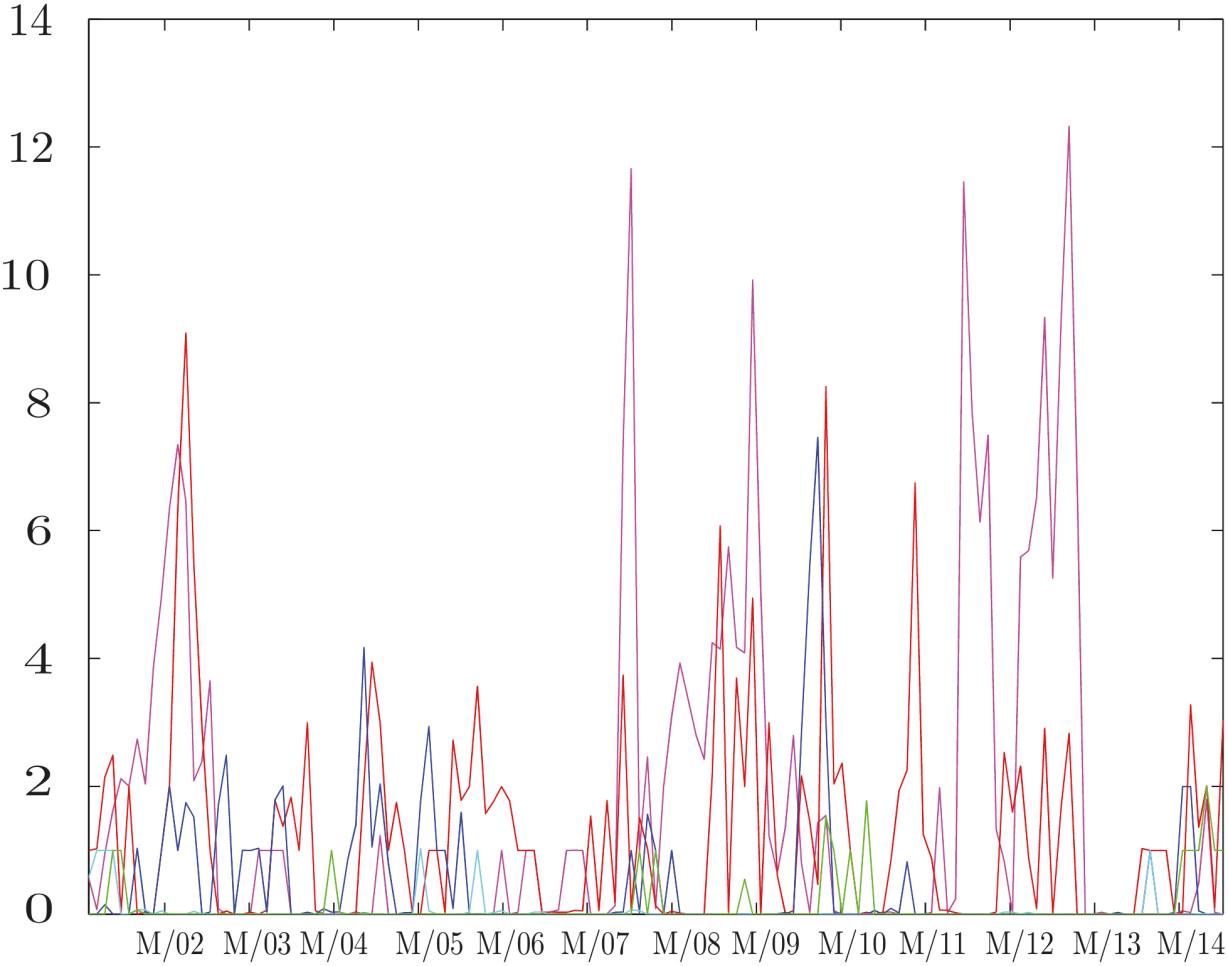
Total information flow as a function of time—The prices time series. Behavior of the total information flow I(w) Eq (6) at different time differences *δ*, when computed for daily prices rather than returns. Each time window *w* is 500 days long. The date associated with each *w* is the middle of the time window considered. The *x*-axis tick marks correspond to the dates of the first of March each year. No meaningful information can be obtained from this analysis in contrast to the results presented in Fig 1.

We repeated the analysis presented in the Result section by using the row prices rather than the returns, even if they are usually not studied in this context. As explained in the Supporting information, there are more or less conservative link extraction protocols, where, roughly speaking, the first ones lead to extract (maybe too) few trustworthy links and the last ones lead to extract (maybe too) many links at the price of considering many spurious links. Employing a less conservative protocol when considering time series of prices we obtained results that resemble those presented above while the exact same protocol used before leads to uninteresting results where no information can be extracted in any window, as shown in Fig 7. This is consistent with the common belief that returns, in general, carry more useful information than prices.

Finally, we would like to comment on the composition of the list of companies considered. As previously stated, the index composition is constantly changing and rather than taking into account these effects we studied the price time series of companies that were part of the index in May 2015. While theoretically the dynamics of the index may be influenced by this choice, the explosion in information flow detected at the onset of the 2008 financial crisis is unlikely to be sensitive to this selection. In fact, as seen in Fig 1, the value of I(w) in 2008 is larger than the background value of the previous year by a factor of about 100. At the same time, the index has a solid backbone, since about 60% of the companies that were part of the index in May 2015 were already included in it in January 2000 [48].

## Conclusions

Market efficiency, or more precisely market unpredictability, has been studied using retarded correlations [27], Granger causality [31] and information theoretical methods [30]. Looking at short time scales, these studies find that log-returns are strongly correlated up to time scales of hours. Longer time periods had been analyzed in [35], using mutual information on daily time series of the closing prices of the components of the US stock market, that was found to be very large during the financial crash of 2008. In our work we develop an information theoretical measure inspired by TE to compute the information flow among daily time series of the closing prices of the components of the FTSE 100 index. Respect to mutual information, TE-like measures are able to detect temporal and directed influences. In fact, they are able to disentangle information exchanged between two inputs from that due to a common driving source, as can be seen in Eq (1). Moreover, they improve on the standard linear methods such as retarded correlations or the Granger causality measure [30, 43], allowing one to extract influences that would be otherwise hardly detectable. Our analysis describes the structure of the market in a long time period spanning from 2000 to 2015, exposing a critical behavior at times of financial crises, as suggested in [35]. Moreover, the analysis supports the dominant viewpoint according to which no information can be extracted at long time scales (of days) at normal day-to-day operations, but sheds light on the emergence of interlinked information flow at times that are close to critical events. We clearly observe that information flow between stocks during non-crises periods have short-lived effects on the market (the market is informationally efficient) whereas during crises they exert their influence over a larger range of time scales, having longer-lasting effects. Near such events the market is thus highly inefficient. Interestingly, we notice that early signals of crashes could already be detected in the markets months before the full manifestation of the crises. The Lehman Brothers bankruptcy, that arguably marks the onset of the 2008 financial crisis, is dated to September 15, 2008, and while this event is clearly recognizable in Fig 1, we also notice that the cross-stock information flow started to grow months in advance, when the American subprime mortgage market started to unfold. Whether or not this method can predict catastrophic events is unclear, but it can definitely measure the susceptibility of financial markets and their robustness to volatility as it exposes the strengthening of long-range correlations, in analogy to complex systems close to a phase transition. The reasons for the inefficiency of financial markets during crashes may have several explanations. The main assumption behind EMH is that traders have rational expectations and that information is easily available to different traders. It is likely that none of the two remain valid during these periods, when herding behavior is known to play a very important role [52]. Moreover, if information were not easily available or if it were processed in different ways by different traders, price movements could become informative and the market become informationally inefficient [33]. According to this point of view, financial crashes can be viewed as collective reorganizations of the market from preexisting equilibria, or possibly out of equilibrium states, to new equilibrium states, taking place on time scales of a few days. The reason why markets may not be at equilibrium before crises, i.e. the reason why prices may not reflect their fundamental values and, thus, markets may not be Pareto efficient, need to be carefully considered. Future work will apply this method to probe information flows at shorter time scales in emergent markets. Looking at finer time scale would provide useful insights in order to characterize the state of markets even far from global crises in weakly efficient markets.

## Supporting information

S1 File Supporting information. Here we give further information about the definition of the transfer entropy, the symbolisation technique and the methods used to filter genuine information from noise.
(PDF)

S1 FigTransfer entropy values—Real and surrogate data.Histograms of values found in the sets T and S for *w* = 80, i.e. the period of November 2008, using the time scale of Fig 1. The inset shows the same quantities computed at *w* = 45, i.e. September 2005. While in the second case no information flows can be detected, in the first, using the protocol discussed in this section, many directed influenced can be obtained. This matrix values refer to *δ* = 2, for which the amount of information is maximized.(EPS)

S2 FigCorrelations between transfer entropy values—Real and surrogate data.Scatter plot of the values found in T at *δ* = 2 in *w* = 80 versus those found in the surrogate dataset at the same *w* and *δ*. The values do not appear to show any correlation between the two.(EPS)
