# Supplementary material for: Emerging interdependence between stock values during financial crashes
Source: PLoS One. 2017 May 25;12(5):e0176764. doi: 10.1371/journal.pone.0176764 (PMC5444585; doi:10.1371/journal.pone.0176764)
Supplement: S1 File — Here we give further information about the definition of the transfer entropy, the symbolisation technique and the methods used to filter genuine information from noise. (PDF) [file pone.0176764.s001.pdf]

Supporting Information

Transfer Entropy

In this section we present the information theoretical tools at the heart of the transfer entropy measure defined in Eq. (1). Let us consider two time series, $X(t)$ and $Y(t)$, and denote by $x_t^{(k)}$ the k past time steps of the time series X at time t: $\{x_{t-k}, \ldots, x_{t-1}\}$. The difference between the probability of observing x_t given $x_t^{(k)}$ and the probability of observing x_t given $x_t^{(k)}$ and $y_t^{(l)}$ can be computed by using the Kullback-Leibler divergence:

$$D_{Y \to X}\left(x_t^{(k)}, y_t^{(l)}\right) = \sum_{x_t} p\left(x_t | x_t^{(k)}, y_t^{(l)}\right) \log\left(\frac{p\left(x_t | x_t^{(k)}, y_t^{(l)}\right)}{p\left(x_t | x_t^{(k)}\right)}\right) . \tag{8}$$

This object is zero if $Y(t)$ contains no information about x_t and positive otherwise. The transfer entropy from $Y(t)$ to $X(t)$, denoted by $T_{Y \to X}$, is the average over the past observations of (8):

$$T_{Y \to X}^{(TE)} = \mathbb{E}_{\{x_t^{(k)}, y_t^{(l)}\}}\left[D_{Y \to X}\left(x_t^{(k)}, y_t^{(l)}\right)\right] . \tag{9}$$

It accounts for the information gained about the present value of the time series $X(t)$ by also considering the l past values of the time series $Y(t)$, in addition to the k past values of $X(t)$. The time steps scale can be generalized from 1 to a general value δ. In this case we have $x_t^{(k)} = \{x_{t-k\,\delta}, \ldots, x_{t-\delta}\}$. Usually, the computation of TE is done by setting $k = l = 1$ for computational reasons; moreover, increasing k may destroy meaningful information flow, as shown in [44].

The computation of TE from the observed time series requires estimation of the various probability distributions in Eq. (9). Among the proposed estimation methods is STE [39], which employs the technique of symbolization. A $k-$dimensional symbol of the time series $X(t)$ at time t is defined by ordering the values $x_{t+\delta}^{(k)} = \{x_{t-(k-1)\delta}, \ldots, x_{t-\delta}, x_t\}$ in an ascending order. The symbol associated with this part of the time series is denoted by $\hat{x}_{t+\delta}^k$. More details on this protocol are given in the next section. The symbolic transfer entropy is then defined by

$$T_{Y \to X}^{(STE)} = \sum_{\hat{x}_{t+\delta}^k, \hat{x}_t^k, \hat{y}_t^k} p\left(\hat{x}_{t+\delta}^k, \hat{x}_t^k, \hat{y}_t^k\right) \log\left(\frac{p\left(\hat{x}_{t+\delta}^k | \hat{x}_t^k, \hat{y}_t^k\right)}{p\left(\hat{x}_{t+\delta}^k | \hat{x}_t^k\right)}\right) , \tag{10}$$

which directly follows from Eq. (9) once that explicit values are replaced by symbols. Assuming stationarity, the required probability distributions can be estimated by computing the occurrences of symbols in the time series, suppressing the effect of noise and bypassing the fine-tuning of parameters in probability distribution estimation protocols. Notice that each symbol is drawn from the values of the time series at k time steps into the past and so that a single symbol contains information from k historic time steps.

The measure we introduced in Eq. (1) is very similar to STE but rather than dealing with k-dimensional symbols, it aims to predict $k+1$-dimensional symbols from k-dimensional ones, but with a modest computational cost. The main reason to introduce this measure has been to gain computational power in predicting symbols of $k+1$ literals; this was particularly important due to the long preprocessing time required for the type of datasets analyzed. The reason is that for each pair of stocks there must be a one-to-one correspondence between the respective trading days. Days

when one of the two is not traded are potentially problematic since they may shift the time index in one of the two and interfere with the causality relations. To deal with this issue we adopted a practical approach by removing all the non-common days in each pair of the time series considered. Since the number of disregarded days in each pair of stocks does not exceed 10 days this may seems a minor difficulty. By the way it requires a larger pre-processing effort, since we cannot symbolize the time series once and for all before computing the matrix T. Instead, we have to pre-process the time series of the stocks on a pair-by-pair basis before symbolizing it for each pair in a dedicated manner, which slows down the process considerably.

Symbolization

Here we provide further details on the symbolization technique. A $k-$dimensional symbol of the time series $X(t)$ at time t,

$$\hat{x}^{k,\delta}_{t+\delta} = \{j_1, \dots, j_{k-1}, j_k\} , \tag{11}$$

is defined by ordering the values $x^{(k)}_{t+\delta} = \{x_{t-(k-1)\delta}, \dots, x_{t-\delta}, x_t\}$ in an ascending order $\{x_{t-(j_1-1)\delta}, \dots, x_{t-(j_{k-1}-1)\delta}, x_{t-(j_k-1)\delta}\}$. If there are repeated values, the one with the smaller index comes first [39]. Here we are going to give a few examples, making the dependance on the time steps scale δ explicit. Let us consider the time series in Table 1.

Table 1

$X(t)$	13	22	45	60	12	33	70	19	20	15	12	42
t	1	2	3	4	5	6	7	8	9	10	11	12

Following the definition given above, and in a way of demonstration, we provide some of the symbols constructed from this time series in Table 2. This table contain samples of symbols extracted from the sequence in Table 1. The first three symbols have $k = 2$, the last three have $k = 3$. For each case we evaluate three different time scales $\delta = 1, 2, 3$.

Table 2

**Symbolization**			
	Symbol	Sequence considered	Symbol value
$k = 2$	$\hat{x}^{2,1}_{12}$	$\{15, 12\}$	$\{2, 1\}$
	$\hat{x}^{2,2}_{11}$	$\{70, 20\}$	$\{2, 1\}$
	$\hat{x}^{2,3}_{10}$	$\{60, 70\}$	$\{1, 2\}$
$k = 3$	$\hat{x}^{3,1}_{12}$	$\{20, 15, 12\}$	$\{3, 2, 1\}$
	$\hat{x}^{3,2}_{11}$	$\{12, 70, 20\}$	$\{1, 3, 2\}$
	$\hat{x}^{3,3}_{10}$	$\{13, 60, 70\}$	$\{1, 2, 3\}$

Details on the evaluation of $I(X, Y)$

In this section we provide further details on the method used to evaluate the values $I(X, Y)$, used in Eqs. (5), (6) and (7). These quantities reflect the amount of genuine information flow from time series $X(t)$ to the time series $Y(t)$ and are obtained by processing the measure introduced in Eq. (1). Cleaning these matrices from spurious values is not an easy task; after the construction of a null model, one needs to employ a thresholding method to filter out random effects.

For each window w, we consider the set $\mathcal{T}$ of TEs from the true dataset and we formed a benchmark set $\mathcal{S}$ of TEs by collecting the values obtained from the surrogate datasets in the 21 windows bracketing w, i.e. $\{w-10, \ldots, w, \ldots, w+10\}$. In other words, we assumed that the null models of consecutive time windows do not differ too much, given that the two windows are shifted by 25 days, corresponding to 5% of their length. A comparison of the histograms $h_T(x)$ and $h_S(x)$ of the set $\mathcal{T}$ and $\mathcal{S}$ gives a crude estimations of the p-values of the TEs computed for the true dataset, i.e. of the probability that the values $x = T_{X \to Y}$ obtained for the true dataset has been obtained at random. This can be done computing, for each x, the ratio

$$r(x) = \frac{\int_x^\infty dx'\, h_S(x')}{\int_x^\infty dx'\, h_T(x')} \,. \tag{12}$$

The ratio r decreases to 0 as x increases: small r values are associated with x values for which it is more likely to have a genuine information flow. Thus, we associated a weigh to each pair $\{X \to Y\}$ given by Eq. (4), that we report below for convenience:

$$I(X,Y) = \frac{1}{e^{2a(r(x)-r^*)}+1} \,, \tag{13}$$

with $x = T_{X \to Y}$, $a = 100$ and $r^* = 0.03$. These two histograms can be seen in S1 Fig. for two particular time windows. One of the possible pitfalls of this method is that values in $\mathcal{T}$ are correlated to values in $\mathcal{S}$. If this were to be the case, we would underestimate the number of detected influences; however, as the scatterplot in S2 Fig. shows, this is not the case. Using this value of r^* can be seen as a soft thresholding method, which roughly corresponds to considering a p-value smaller than 0.05. Statistically validated networks are obtained by considering much smaller thresholds that take into account multiple comparison effects. By the way, employing such a strict protocol would provide poor results in the present case because the possible retrievable information is very small and we are forced to adopt a less conservative protocol. Nevertheless, our results have been cross-validated by using the method described in section and the fact that the null model is unable to reproduce the total information flow patterns detected in the original dataset validates the quality of the analysis.

S1 Fig. Transfer entropy values - real and surrogate data. Histograms of values found in the sets $\mathcal{T}$ and $\mathcal{S}$ for $w = 80$, i.e. the period of November 2008, using the time scale of Fig. 1. The inset shows the same quantities computed at $w = 45$, i.e. September 2005. While in the second case no information flows can be detected, in the first, using the protocol discussed in this section, many directed influenced can be obtained. This matrix values refer to $\delta = 2$, for which the amount of information is maximized.

S2 Fig. Correlations between transfer entropy values - real and surrogate data. Scatter plot of the values found in $\mathcal{T}$ at $\delta = 2$ in $w = 80$ versus those found in the surrogate dataset at the same w and δ. The values do not appear to show any correlation between the two.
